# Ring-Selective Fragmentation in the Tirapazamine Molecule upon Low-Energy Electron Attachment

**DOI:** 10.3390/ijms22063159

**Published:** 2021-03-19

**Authors:** Eugene Arthur-Baidoo, Joao Ameixa, Milan Ončák, Stephan Denifl

**Affiliations:** 1 Institute for Ion Physics and Applied Physics, University of Innsbruck, Technikerstrasse 25/3, 6020 Innsbruck, Austria; Eugene.Arthur-Baidoo@uibk.ac.at (E.A.-B.); j.ameixa@campus.fct.unl.pt (J.A.); 2 Center for Molecular Biosciences Innsbruck, University of Innsbruck, Technikerstrasse 25/3, 6020 Innsbruck, Austria; 3 Atomic and Molecular Collisions Laboratory, Department of Physics, CEFITEC, Universidade NOVA de Lisboa, 2829-516 Caparica, Portugal

**Keywords:** radiosensitizer, low-energy electron, dissociative electron attachment, roaming, tirapazamine, predissociation

## Abstract

We investigate dissociative electron attachment to tirapazamine through a crossed electron–molecule beam experiment and quantum chemical calculations. After the electron is attached and the resulting anion reaches the first excited state, D_1_, we suggest a fast transition into the ground electronic state through a conical intersection with a distorted triazine ring that almost coincides with the minimum in the D_1_ state. Through analysis of all observed dissociative pathways producing heavier ions (90–161 u), we consider the predissociation of an OH radical with possible roaming mechanism to be the common first step. This destabilizes the triazine ring and leads to dissociation of highly stable nitrogen-containing species. The benzene ring is not altered during the process. Dissociation of small anionic fragments (NO_2_^−^, CN_2_^−^, CN^−^, NH_2_^−^, O^−^) cannot be conclusively linked to the OH predissociation mechanism; however, they again do not require dissociation of the benzene ring.

## 1. Introduction

Attempts to find innovative methods for applications in cancer radiotherapy and concomitant chemotherapy have been a focus of research for decades. Hypoxic cells in solid tumors, however, pose resistance to radiotherapy, resulting in a reduced tumor response [1,2,3]. The chemical agents, referred to as radiosensitizers, administered during cancer radiotherapy have the capability of differentially sensitizing hypoxic cells and thus enhancing radiation-induced DNA damage, especially in the local tumor regions [4,5]. Therefore, new radiosensitizing agents are still being suggested and investigated to counter this effect of radiation resistance in hypoxic regions [6].

Hypoxic cytotoxins are a kind of radiosensitizer designed to only activate under hypoxic conditions [7]. Their ability to accumulate and retain their integrity in tumor cells under low oxygen content contributes greatly to their radiosensitization property. Usually, such compounds can undergo a reduction process, converting them into reactive radicals, which are able to accumulate in hypoxic tumor cells and, subsequently, induce DNA damages [5]. Electron attachment studies with nimorazole [8], carried out in the gas phase and in the microsolvated conditions, endorse the hypothesis that the protonated radical anion of this hypoxic radiosensitizer [9] mimics the action of molecular oxygen.

One compound which has demonstrated its potential in terms of highly selective cytotoxicity is the heterocyclic–n–oxide derivative, 3–amino 1,2,4–benzotriazine 1,4–dioxide or tirapazamine (TPZ) [6,10]. Under hypoxic conditions, the antitumor prodrug TPZ is transformed into a radical intermediate TPZ^•−^ via a one-electron reduction process that, in a second step, becomes protonated, TPZH^•^ [5,11]. The next reaction steps, which yield bioactive radicals considered to be the precursors for DNA damage, are still under debate. A few studies [12,13,14] suggested the release of OH^•^ radicals from the protonated radical anion TPZH^•^, while others proposed the formation of the radical benzotriazinyl, BTZ^•^ upon loss of neutral water from TPZH^•^ [15,16]. Then, DNA double-strand breaks arise as the enzyme topoisomerase II gets inactivated by one of these bioactive radicals, i.e., OH^•^ or BTZ^•^ [11]. In the presence of oxygen, the radical intermediate, TPZ^•−^ is, however, back-oxidized into the nontoxic parent compound with the release of reactive oxygen species (ROS) as a byproduct [17]. Early studies suggested that TPZ can act as a substitute for molecular oxygen (O_2_) in the conversion of DNA radicals into toxic strand damage [18,19].

It has been established that the radiosensitization ability of chemical sensitizers is dependent on their affine nature toward electrons [20] and the subsequent production of free radicals [21]. Low-energy electrons (LEEs), with an energy distribution peaking at about 10 eV, are released in the order 10^4^/MeV upon the impact of ionizing radiation with biological tissue [21]. Water radiolysis leads to the formation of LEEs, as well as ROS in the physical and physicochemical stages of radiation damage (<10^−12^ s). LEEs may become hydrated in the chemical stage (<10^−6^ s) [22,23], or, if formed near a biological DNA system, they induce damage in DNA via bond cleavage, primarily driven by dissociative electron attachment (DEA) [23,24,25].

Low-energy electron attachment to TPZ has been studied recently [26]. In summary, this study showed the formation of the intact parent anion (TPZ)^−^, the dehydrogenated parent anion (TPZ–H)^−^, the fragment anion due to loss of NH_2_ forming (TPZ–NH_2_)^−^ via single bond cleavage and the loss of the hydroxyl radical to form the most abundant fragment anion (TPZ–OH)^−^ with mass of 161 u. To account for the mechanism of dissociative electron attachment, we proposed a roaming mechanism in which an OH moiety is formed in the first step and then either evaporates or roams in the vicinity of the molecule to react with various parts of the (TPZ–OH)^−^ core. The roaming mechanism is expected to be suppressed with solvation. Single-reference quantum chemical calculations showed that the ground state potential energy surface (PES) of TPZ might cross the PES of the first excited state in TPZ^−^, explaining the resonance near 0 eV. In another study using density functional calculations, Yin et al. [27] showed some possible pathways for reduced TPZ species after the N4-OH homolytic processes. It was reported that the cleavage of C-N bonds leads to ring opening by bond rupture in the triazine moiety [27,28].

Herein, we investigate additional fragmentation pathways of tirapazamine upon low-energy electron interaction in the energy range of 0–12 eV. We report ten further fragmentation channels attributed to multiple bond cleavages in the parent molecule. It is worth noting that, regardless of the dissociations observed in TPZ, the integrity of the benzene structure is preserved. All fragmentation pathways are attributed to the dissociation of the triazine moiety attached to the benzene ring.

## 2. Results

The DEA process may be the underlying physico-chemical mechanism for the action of some potential radiosensitizers like modified nucleobases [29,30,31] and for the radiation damage of molecules of biological relevance, like nucleobases, amino acids, etc. [32,33,34]. The mechanism of dissociation is described to proceed via Equation (1):

e^−^ + M → M*^−^ → (M − X) + X^−^ (1)

The incoming electron is captured by the parent molecule M yielding a transient negative ion (TNI), M*^−^, also termed resonance, which subsequently dissociates into a neutral fragment (M–X) and a fragment anion X^−^. The superscript * indicates that the TNI may be formed in an excited state [33,34].

Before we discuss dissociation pathways of TPZ^−^, we focus on the events that take place directly after electron attachment. As the vertical electron affinity of TPZ is about 1.3 eV, the formed TPZ*^−^ anion is prepared in an electronically excited state. Our time-dependent density functional theory (TDDFT) calculation at the TD–BMK/aug–cc–pVDZ level shows that the first excited state of TPZ*^−^, D_1_, lies at 1.65 eV, i.e., it is energetically well-separated from the ground electronic state. The first ten excited states are relatively densely packed within 1.65–3.30 eV (see Table S1). We can thus conclude that, in any electronic state reached after electron attachment, one expects that the system switches readily to the D_1_ state through nonradiative processes, due to the low energy separation between the excited states (Kasha’s rule).

**Figure 1:**
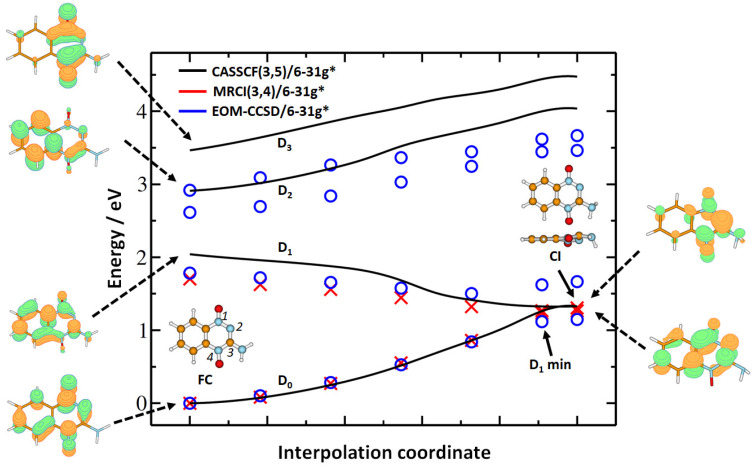
Interpolation from the Franck–Condon point (FC) to the minimum on the D_1_ electronic state potential energy surface in TPZ^−^ and further to the D_0_/D_1_ conical intersection (CI). The FC point was optimized at the B3LYP/aug–cc–pVDZ level of theory, D_1_ minimum and CI at the complete active space–self consistent field (CASSCF)(3,5)/6–31g* level. The interpolation points were recalculated at the multireference configuration interaction (MRCI)(3,4) and equation of motion-coupled clusters singles and doubles (EOM-CCSD) level with the 6–31g* basis set. For the CI, both top and side view is provided to show the triazine ring distortion. On the left-hand and right-hand sides, singly occupied orbitals, according to the configuration with the highest contribution to the respective electronic state in the configuration interaction vector, are shown as calculated in the FC point and CI at the CASSCF level.

Figure 1 analyzes the fate of the TPZ*^−^ anion in the D_1_ state. Note that a smaller basis is used for calculations so that the excited states are not that close to each other, as in the calculation with the aug–cc–pVDZ basis set. When we optimize the molecule in the D_1_ state starting from the planar Franck–Condon (FC) structure, a minimum with a considerably distorted triazine ring is reached. This minimum almost coincides with the D_0_/D_1_ conical intersection (CI), through which the system might funnel to the ground electronic state; in the CI structure, the benzene ring stays almost planar, while the triazine ring shows the N-N-C-N dihedral angle of 30.3° and a pyramidalized NH_2_ group. Thus, when the TPZ*^−^ anion enters the D_1_ state (either through direct electron attachment or after passing from a higher electronic state), the D_0_/D_1_ conical intersection can be reached without overcoming any barrier, and we can expect the switch from D_1_ to D_0_ on the order of hundreds of femtoseconds. In the ground state, we expect that TPZ^−^ regenerates its FC structure, as the CI structure with a deformed triazine ring does not clearly hint toward any dissociation pathway.

From the methodological perspective, we can see that, when accounting for dynamical correlation through either multireference configuration interaction (MRCI) or equation of motion-coupled clusters singles and doubles (EOM-CCSD) method, the excitation energies in the FC point shift to lower values, compared to the complete active space–self consistent field (CASSCF) ones. Higher lying states are not well described by the CASSCF method, due to limited active space. The character of the conical intersection is maintained at the MRCI level, with the difference between D_0_ and D_1_ states of 0.04 eV on the MRCI(3,4)/6–31g*//CASSCF(3,5)/6–31g* level. Calculations at the MRCI and EOM-CCSD levels are very close to each other in the FC point and at the beginning of the interpolation pathway; when D_0_ and D_1_ electronic states approach each other, the single-reference EOM-CCSD method is unable to retrieve the potential energy surface of D_1_ reliably. Within the single-reference TD–BMK method, a D_1_ minimum of a similar structure but with a considerable D_0_/D_1_ gap is thus obtained [26]. Finally, the shift from the 6–31g* basis set to aug–cc–pVDZ within the TD–BMK approach has a very limited effect on the position of valence states included in Figure 1 in the FC point (with a difference in energy below 0.04 eV).

**Figure 2:**
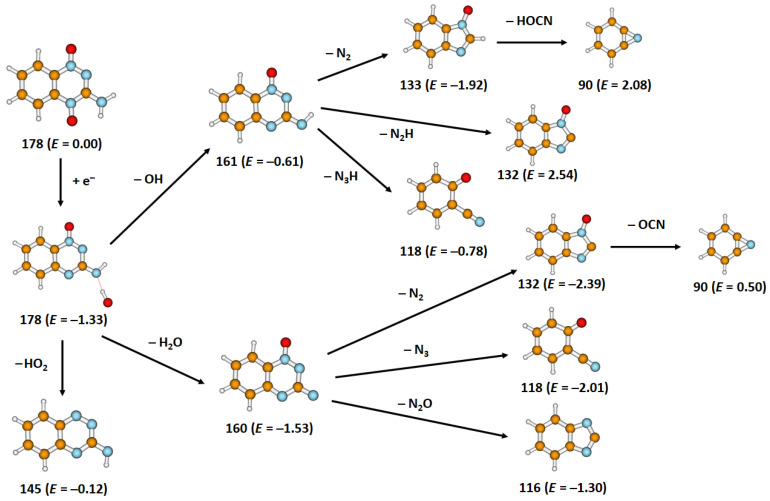
Suggested fragmentation pathways after electron attachment to tirapazamine starting with predissociation of the OH moiety; the mass (u) and energy *E* (in eV) of the respective anions are given. Calculated at the B3LYP/aug–cc–pVDZ level of theory.

Further, we focus on the fragmentation mechanisms, resonances positions for the detected anions, experimental onsets of the various anions and quantitatively determined thermodynamic thresholds, as presented in Table 1. The mechanisms leading to the formation of the fragment anions (TPZ–H)^−^, (TPZ–NH_2_)^−^ and (TPZ–OH)^−^ included in Table 1 were analyzed previously [26]. The suggested reaction pathways for ions resulting from initial OH predissociation are shown in Figure 2.

**Figure 3:**
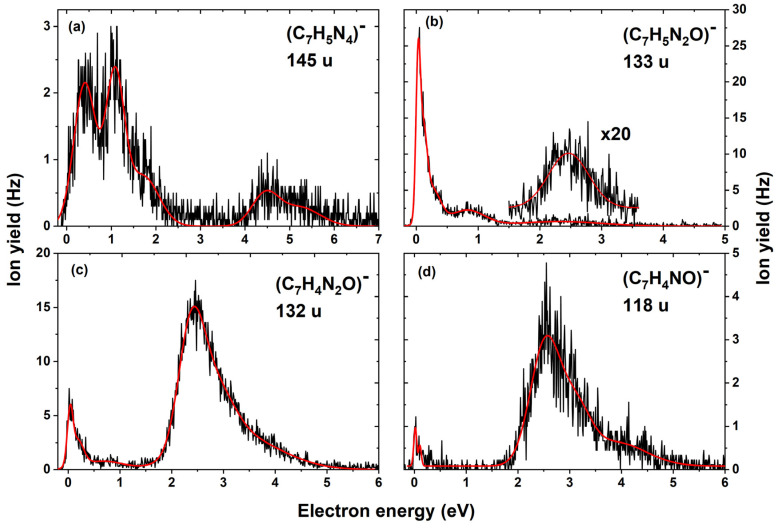
Anion efficiency curves for the formation of the anion with masses (**a**) 145 u, (**b**) 133 u, (**c**) 132 u and (**d**) 118 u upon electron attachment to tirapazamine. The solid red line is a cumulative sum of individual gaussian functions rendering the observed features.

Figure 3a shows the anion efficiency curve for the formation of C_7_H_5_N_4_^−^. We observed a first resonance region below 2 eV, with maxima at 0.4, 1.0 and 1.7 eV. A second resonance region was observed, but with low intensity, between 4 and 6 eV, with a peak maximum of around 4.5 and 5.3 eV. The formation of C_7_H_5_N_4_^−^ with the mass of 145 u is attributed to the loss of a hydroxyl group and oxygen at the 1–N–oxide position, see Equation (2). We expect that the anion results from the OH roaming mechanism. During the process, the roaming OH radical attaches to the O atom and dissociates as HO_2_.

C_7_H_6_N_4_O_2_ + e^−^ → (C_7_H_6_N_4_O_2_)^−*^ → (C_7_H_5_N_4_O…OH)^−*^ → C_7_H_5_N_4_^−^ + HO_2_ (2)

The calculated thermodynamic threshold predicts an exothermic reaction with a threshold of −0.12 eV, in agreement with the observed experimental onset of 0 eV. The channel is thermodynamically less favorable than direct OH dissociation (see Figure 2), and the OH radical can evaporate before it reaches the oxygen atom, which is reflected in its low contribution to the total ion yield. Note that subsequent dissociation of OH and O would lead to a too high reaction energy. Both rings stay intact during the dissociation process.

Equations (3) and (4) show the formation of the fragment anions with masses 133 u and 132 u. These anions are assigned to the C_7_H_5_N_2_O^−^ and C_7_H_4_N_2_O^−^ structures, respectively.

C_7_H_6_N_4_O_2_ + e^−^ → (C_7_H_6_N_4_O_2_)^−*^ → C_7_H_5_N_2_O^−^ + OH + N_2_ (3)

C_7_H_6_N_4_O_2_ + e^−^ → (C_7_H_6_N_4_O_2_)^−*^ → C_7_H_4_N_2_O^−^ + H_2_O + N_2_ (4)

From the anion efficiency curve shown in Figure 3b, we derived four main resonances for C_7_H_5_N_2_O^−^ (mass 133 u), with peak maxima comprising a sharp peak at 0.0 eV; low-intensity peaks at 0.1 eV, 0.3 eV, 0.8 eV and a broad resonance around 2.5 eV. Comparing Figure 3b,c, similar resonance positions are observed below 2 eV region for C_7_H_4_N_2_O^−^ (mass 132 u) with a lower intensity. In addition, the asymmetric shape of the main peak in the ion yield of C_7_H_4_N_2_O^−^ (see Figure 3c) suggests resonances at 2.8 eV and 3.2 eV. On the other hand, in the energy range of 1.5–5 eV, the intensity of C_7_H_4_N_2_O^−^ prevails over the one of C_7_H_5_N_2_O^−^ by an order of magnitude.

For both anions, we suggest that the reaction starts with predissociation of the OH moiety (Figure 2), as supported by the fact that the resonance positions below 1 eV for the formation of these anions are exactly at the same positions [26]. This implies that both result from the same TNI.

For C_7_H_5_N_2_O^−^, we associate the formation of the anion with the loss of a hydroxyl group, followed by a loss of N_2_ upon an initial opening of the triazine ring. The final structure has two closed rings, formed through structural rearrangement, as shown in Figure 2. For C_7_H_4_N_2_O^−^, we expect a water molecule to be formed through the roaming mechanism, again with a subsequent N_2_ dissociation (Figure 2). For electron energies above 2.54 eV (as calculated at the B3LYP/aug–cc–pVDZ level), this ion may be also formed through dissociation of OH + N_2_H. This might explain the prevalence of C_7_H_4_N_2_O^−^ over C_7_H_5_N_2_O^−^ for higher electron energies.

The dissociation of N_2_ or N_2_H requires fundamental rearrangement of the triazine moiety; however, a considerable amount of energy is gained during the process that allows for subsequent dissociation processes (see discussion of the C_6_H_4_N^−^ anion with mass 90 u below). The exothermicity of the reactions agrees with the experiment. The low yield of the subsequent dissociation reaction can be explained by energetic considerations: only if the OH radical does not take too much kinetic energy away from the system, the remaining anion can surpass a barrier for ring dissociation.

Figure 3d shows the anion efficiency curve for mass 118 u with three resonance regions, one near 0 eV, a peak with maximum around 2.5 eV followed by resonances centered at 3.2 and 3.9 eV. For this anion, two different plausible assignments are possible, C_7_H_6_N_2_^−^ and C_7_H_4_NO^−^. In the first case, N_2_O_2_ would dissociate. Our calculations show that the only exothermic reaction for this dissociation would be dissociation of N_2_ + O_2_ with the reaction energy of −0.77 eV, which does not seem reasonable from the kinetic perspective. The C_7_H_4_NO^−^ ion might be formed through dissociation of [N_3_OH_2_], suggesting either dissociation of OH + N_3_H or H_2_O + N_3_, Equations (5a) and (5b) and implying, again, the OH predissociation as the initial step (Figure 2).

C_7_H_6_N_4_O_2_ + e^−^ → (C_7_H_6_N_4_O_2_)^−*^ → C_7_H_4_NO^−^ + OH + N_3_H (5a)

C_7_H_6_N_4_O_2_ + e^−^ → (C_7_H_6_N_4_O_2_)^−*^ → C_7_H_4_NO^−^ + H_2_O + N_3_ (5b)

Both Equations (5a) and (5b) are exothermic (−0.78 and −2.01 eV). Similarities of the peak positions in the anion efficiency curves of C_7_H_5_N_2_O^−^, C_7_H_4_N_2_O^−^ and C_7_H_4_NO^−^ support our interpretation of the molecular mechanism.

**Figure 4:**
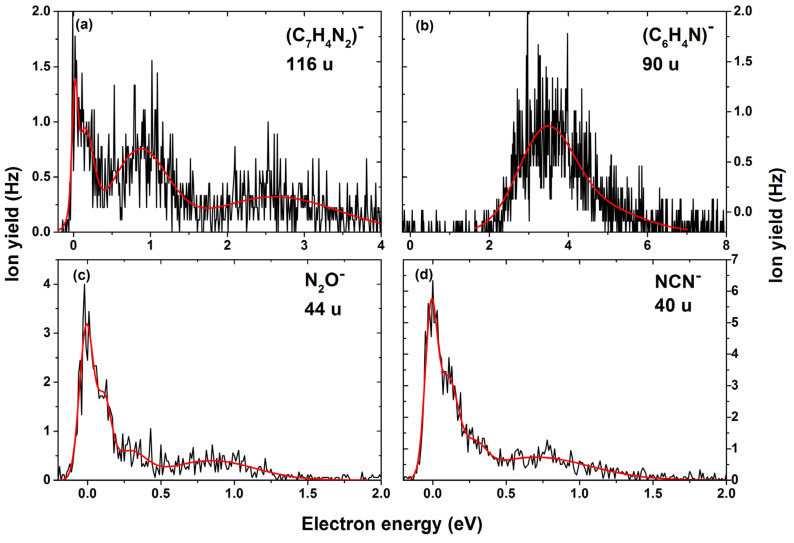
Anion efficiency curve for the formation of the anions with masses (**a**) 116 u, (**b**) 90 u, (**c**) 44 u and (**d**) 40 u upon electron attachment to tirapazamine.

We also observed a low-intensity anion with mass 116 u. The corresponding anion efficiency curve shows resonance positions that were observed previously for (TPZ–OH)^−^ formation (0.0, 0.1, 0.9 eV); see Figure 4a. A further broad resonance with a maximum at about 2.6 eV is also present in the ion yield. We interpret this channel as formation of C_7_H_4_N_2_^−^ arising due to the loss of H_2_O and N_2_O upon multiple bond cleavages and rearrangement in the parent molecule; see Equation (6) and Figure 2. The predicted reaction energy of −1.30 eV agrees with the experimental onset.

C_7_H_6_N_4_O_2_ + e^−^ → (C_7_H_6_N_4_O_2_)^−*^ → C_7_H_4_N_2_^−^ + H_2_O + N_2_O (6)

Figure 4b shows the anion efficiency curve for the formation of the anion with mass 90 u, assigned to C_6_H_4_N^−^. We observed one broad bump with a maximum at about 3.6 eV and a second resonance at 4.7 eV near the tail. We suggest that this ion emerges in a follow-up reaction of Equations (3) and/or (4):

C_7_H_6_N_4_O_2_ + e^−^ → (C_7_H_6_N_4_O_2_)^−*^ → C_6_H_4_N^−^ + OH + N_2_ + HOCN (7a)

C_7_H_6_N_4_O_2_ + e^−^ → (C_7_H_6_N_4_O_2_)^−*^ → C_6_H_4_N^−^ + H_2_O + N_2_ + OCN (7b)

From the thermodynamic perspective, the first reaction is considerably less probable (2.08 eV vs. 0.50 eV). The anion is, however, observed only at higher energies and with low intensity; the experimental onset at 2.1 eV suggests that C_6_H_4_N^−^ is formed upon Equation (7a), though we point out that Equation (7b) would be thermodynamically open, as well. 

Our analysis shows that the formation mechanisms for the anions with masses 90–161 u have several common features: (1) The first step in their formation is predissociation of the OH group formed after proton transfer from the NH_2_ group to the adjacent O atom. (2) The OH group dissociates either alone, after another proton transfer (forming H_2_O) or as HO_2_ (minor channel). (3) Highly stable nitrogen-containing species seem to dissociate in the subsequent step. (4) The benzene ring stays intact, while the triazine part of the molecule undergoes significant changes. It is also interesting to note that (TPZ–H_2_O)^−^ with mass 160 u, corresponding to water dissociation, is not observed in the experiment. One of the reasons could be the high energy release (−1.53 eV), enabling further dissociation reactions.

Although the energy minima presented in Figure 2 do not provide direct hints toward dissociation kinetics, one should keep in mind the high energy content in molecules (with three exceptions, all reactions depicted in Figure 2 are exothermic). Molecular dynamics runs of (TPZ–OH)^−^ at elevated temperature show that the bonds in the triazine moiety are the weakest ones and might break preferentially.

**Figure 5:**
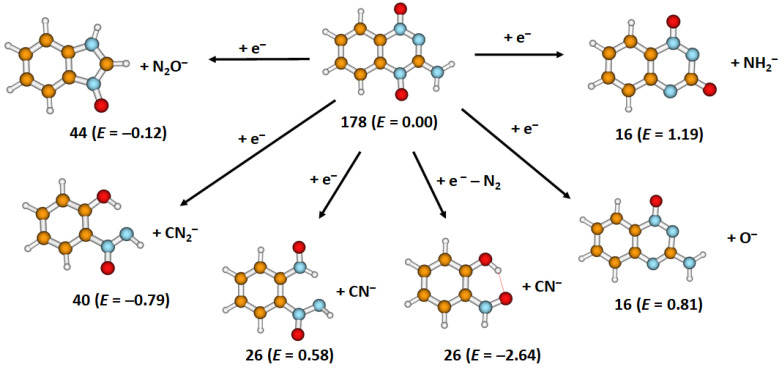
Suggested fragmentation pathways producing small anions after electron attachment to tirapazamine; the mass (in u) and energy *E* (in eV) of the respective anions are given. Calculated at the B3LYP/aug–cc–pVDZ level of theory.

Finally, we focus on the formation of small negative ions that can be formed through dissociation of the triazine moiety (Figure 5). Note that the calculated dissociation energies are conservative estimates as, if one allows for further dissociation of the molecule through, e.g., formation of N_2_ molecules, the dissociation energy drops significantly. At the same time, it is possible to connect the dissociation pathways to the predissociation of the OH group; however, this is not needed from the thermodynamical perspective.

Figure 4c,d show the anion efficiency curve for the formation of N_2_O^−^ (mass 44 u) and CN_2_^−^ (mass 40 u) due to multiple bond cleavage in the benzotriazine dissociation upon electron attachment to tirapazamine, via Equations (8) and (9), respectively. Both anions show resonance positions at 0, 0.1, 0.3 eV and a broad peak around 0.8 eV close to the tail. The calculated reaction energy is markedly exothermic for both N_2_O^−^ (−0.12 eV) and CN_2_^−^ (−0.79 eV) (see Figure 5).

C_7_H_6_N_4_O_2_ + e^−^ → (C_7_H_6_N_4_O_2_)^−*^ → N_2_O^−^ + C_7_H_6_N_2_O (8)

C_7_H_6_N_4_O_2_ + e^−^ → (C_7_H_6_N_4_O_2_)^−*^ → CN_2_^−^ + C_6_H_6_N_2_O_2_ (9)

**Figure 6:**
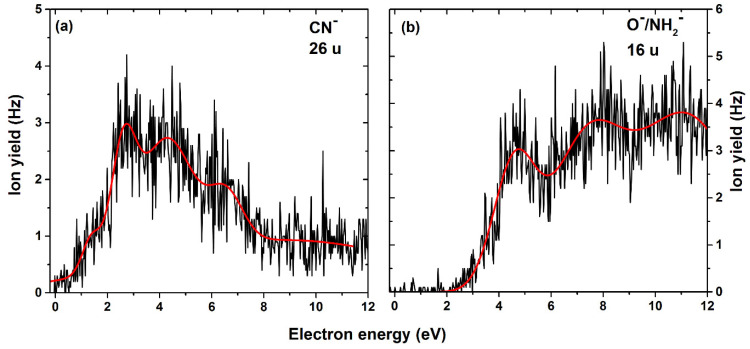
Anion efficiency curve for the formation of (**a**) CN^−^ and (**b**) O^−^/NH_2_^−^ upon electron attachment to TPZ.

In the anion efficiency curve for the anion with mass 26 u, we observe few overlapping resonances (Figure 6a). We assign this ion yield to CN^−^; the alternative explanation of C_2_H_2_^−^ seems less probable, due to the need to break the benzene ring that is not observed for any other fragment. Like the formation of CN^−^ in nitroimidazoles [35], the anion is formed via an exothermic Equation (10) only after a rupture of the triazine side.

C_7_H_6_N_4_O_2_ + e^−^ → (C_7_H_6_N_4_O_2_)^−*^ → CN^−^ + C_6_H_6_N_3_O_2_ (10a)

C_7_H_6_N_4_O_2_ + e^−^ → (C_7_H_6_N_4_O_2_)^−*^ → CN^−^ + C_6_H_6_NO_2_ + N_2_ (10b)

While the simple dissociation of CN^−^ is still endothermic by 0.58 eV, the reaction energy drops to −2.64 eV if N_2_ is dissociated at the same time.

We also observed, as the smallest of all fragments, the anion with mass 16 u, which could be assigned to either O^−^ or NH_2_^−^, since they have the same nominal mass. The separation of isobaric anions requires high-resolution mass spectrometers, which is a limitation in our present experimental setup, and therefore, we are not able to distinguish the two isobaric anions. Three high-energy resonances with peak maxima at 4.6, 7.3 and 11.1 eV are observed; see Figure 6b. The reactions leading to the formation of this anion yield are predicted to take place through direct dissociation (Figure 5) and are endothermic, with 0.81 eV and 1.19 eV for dissociation of O^−^ and NH_2_^−^, respectively. Therefore, at the threshold determined experimentally of 2.7 eV, the formation of both anions O^−^ and NH_2_^−^ is thermodynamically possible.

## 3. Materials and Methods

Tirapazamine (C_7_H_6_N_4_O_2_) was purchased from Sigma Aldrich (Vienna, Austria) with a purity of ≥ 98% (HPLC) and was used as received. The experiment was performed in the gas phase under a high vacuum (10^−8^ mbar) using a crossed electron–molecule beam apparatus previously described in [36]. Briefly, the setup consists of a homemade hemispherical electron monochromator (HEM), a resistively heated copper oven, a quadrupole mass analyzer (QMA 400, Pfeiffer Vacuum, Vienna, Austria) and a detector for counting the mass-analyzed ions. The neutral beam tirapazamine in the gas phase was produced by heating the sample in the oven and expanding the vapor through a 1 mm diameter capillary. Then, the effusive molecular beam was crossed perpendicularly with the electron beam. An electron beam with energy resolution of 100 meV at FWHM (full width at half maximum) and current of 8–19 nA was used, ensuring a compromise between resulting ion beam intensity and the electron energy resolution. Before starting the measurements of negative ions, the temperature dependency of the electron ionization mass spectrum was checked to obtain a reasonable ion signal without decomposition of the sample in the oven. The compound was finally studied at the oven temperature of 395 K. The ions formed upon (dissociative) electron attachment were extracted to the QMA entrance by a weak electrostatic field, mass-analyzed in the QMA and detected by a channeltron secondary electron multiplier operated in single-pulse counting mode. The anion efficiency curves were recorded by a scan of the electron energy, while the QMA was set for transmission of a chosen anion. For calibration of the electron energy scale and determination of the energy resolution, the well-known Cl^−^/CCl_4_ resonance at 0 eV [37] was used. The experimental threshold for the detected DEA reactions with TPZ was determined with the method proposed in [38].

Molecular structures were optimized at the B3LYP/aug–cc–pVDZ level of theory. Wavefunction stability was checked in every point, and all reported reaction energies include zero-point corrections. To investigate excited states of the anion formed after electron attachment, we considered single-reference methods, time-dependent density functional theory (TDDFT) and equation of motion-coupled clusters singles and doubles (EOM-CCSD) [39,40,41], as well as multireference approaches, complete active space–self consistent field (CASSCF) and multireference configuration interaction (MRCI). For CASSCF calculations, the active space of 3 electrons in 5 orbitals (further denoted as (3,5)) was chosen for TPZ^−^ to cover the most important, low-lying valence electronic states. We employed averaging over four electronic states. MRCI calculations were performed in the (3,4) active space, coring 20 lowest-lying orbitals. To prevent electronic states of diffuse character from appearing, we limited ourselves to the 6–31g* basis set in multireference calculations. We tested the suitability of the approximations involved (limited active space, a small basis set) through comparison to the results of single-reference calculations in the Franck–Condon (FC) point. While our calculations rationalize the resonance at 0 eV (with D_1_ as the target state), more advanced methods would be needed to reproduce positions of higher-lying resonances. For the multireference calculations, we employed the Molpro package [42], all other calculations were performed in the Gaussian program [43].

## 4. Conclusions

In the present publication, we analyzed processes after electron attachment to TPZ, as well as induced dissociation pathways. The ground electronic state of TPZ^−^ is readily reached through a D_0_/D_1_ conical intersection that has a distorted triazine ring and almost coincides with the D_1_ minimum. We showed that the crucial step in the dissociation kinetics of all anions containing the benzene ring (anions with masses 90–161 u) is the predissociation of an OH group that roams in the anion vicinity, dissociating either directly or after forming HO_2_/H_2_O. When the triazine part of the molecule is destabilized by dissociation of at least one oxygen atom, degradation of the ring continues through dissociation of highly stable species with at least two nitrogen atoms. The extent of decomposition will depend on the energy carried away by the dissociating O*_x_*H*_y_* fragment. The benzene ring, on the other hand, plays the role of a spectator and is not altered during the dissociation process. Similarly, all small anionic species are dissociated from the triazine part of the molecule, with the benzene ring left intact. Formation of N_2_O^−^, CN_2_^−^ and CN^−^ anions might also take place after OH group dissociation; our data, however, do not allow for any conclusion in this respect.

## Figures and Tables

**Table 1:** Summary of anions observed upon electron attachment to tirapazamine, along with their mass, suggested molecular structure, associated neutral fragment(s), resonance positions, experimental threshold and thermodynamically determined threshold. Energies were calculated at the B3LYP/aug–cc–pVDZ level of theory; suggested neutral fragments are based on theoretical analysis.

Mass (u)	Anion	Neutral Counterparts		Resonance Positions (eV)						Thresholds (eV)	
			1	2	3	4	5	6	7	Exp	Theory
178	C_7_H_6_N_4_O_2_^−^		0	0.1	0.3	-	-	-	-	~0	−1.28
177 ^a^	C_7_H_5_N_4_O_2_^−^	H	0	0.1	0.3	0.9	-	-	-	~0	−0.92
162 ^a^	C_7_H_4_N_3_O_2_^−^	NH_2_	0.3	0.7	-	-	-	-	-	~0	−1.71
161 ^a^	C_7_H_5_N_4_O^−^	OH	0	0.1	0.3	0.9	-	-	-	~0	−0.61
145	C_7_H_5_N_4_^−^	HO_2_	0.4	1.0	1.7	4.5	5.3	-	-	~0	−0.12
133	C_7_H_5_N_2_O^−^	OH + N_2_	0.0	0.1	0.3	0.8	2.4	-	-	~0	−1.92
132	C_7_H_4_N_2_O^−^	H_2_O + N_2_/ OH + N_2_H	0.0	0.1	0.3	0.8	2.4	2.8	3.2	~0	−2.39/ 2.54
118	C_7_H_4_NO^−^	OH + N_3_H/ H_2_O + N_3_	0.0	0.1	2.5	3.2	3.9	-	-	~0	−0.78/ −2.01
116	C_7_H_4_N_2_^−^	H_2_O + N_2_O	0.0	0.1	0.9	2.6	-	-	-	~0	−1.30
90	C_6_H_4_N^−^	OH + N_2_ + HOCN/H_2_O + N_2_ + NCO	3.6	4.7	-	-	-	-	-	2.1	2.08/ 0.50
44	N_2_O^−^	C_7_H_6_N_2_O	0.0	0.1	0.3	0.9	-	-	-	~0	−0.12
40	NCN^−^	C_6_H_6_N_2_O_2_	0.0	0.1	0.3	0.7	-	-	-	~0	−0.79
26	CN^−^	C_6_H_6_N_3_O_2_/ C_6_H_6_NO_2_ + N_2_	1.4	2.6	4.2	6.5	-	-	-	0.7	0.58/ −2.64
16	O^−^ NH_2_^−^	C_7_H_6_N_4_O C_7_H_4_N_3_O_2_	4.6	7.3	11.1	-	-	-	-	2.7	0.81 1.19

^a^—Dissociation pathways were analyzed previously [26].

## Data Availability

The data presented in this work are available on request from the corresponding authors.

## Supplementary Materials

The following are available online at https://www.mdpi.com/1422-0067/22/6/3159/s1, Table S1. First ten excitation energies (in eV) of TPZ^−^ calculated at the TD-BMK/aug-cc-pVDZ level in the structure of TPZ optimized at the B3LYP/aug-cc-pVDZ level. Cartesian coordinates (in Ångstrom) and electronic energies calculated at the B3LYP/aug-cc-pVDZ level including the zero-point energy (in Hartree).
