# Supplementary material for: Ring-Selective Fragmentation in the Tirapazamine Molecule upon Low-Energy Electron Attachment
Source: Int J Mol Sci. 2021 Mar 19;22(6):3159. doi: 10.3390/ijms22063159 (PMC8003736; doi:10.3390/ijms22063159)
Supplement: Supplementary file 1 [file ijms-22-03159-s001.pdf]

Supporting Information

for

Ring-Selective Fragmentation in the Tirapazamine Molecule upon Low-Energy Electron Attachment

Eugene Arthur-Baidoo[1,2], Joao Ameixa[1,2,3], Milan Ončák[1,*] and Stephan Denifl[1,2,*]

[1] Institute for Ion Physics and Applied Physics, University of Innsbruck, Technikerstrasse 25/3, 6020 Innsbruck, Austria; Eugene.Arthur-Baidoo@uibk.ac.at (E.A.-B.); j.ameixa@campus.fct.unl.pt (J.A); Milan.Oncak@uibk.ac.at (M.O); Stephan.Denifl@uibk.ac.at (S.D.)

[2] Center for Biomolecular Sciences Innsbruck, University of Innsbruck, Technikerstrasse 25/3, 6020 Innsbruck, Austria; Eugene.Arthur-Baidoo@uibk.ac.at (E.A.-B.); j.ameixa@campus.fct.unl.pt (J.A); Stephan.Denifl@uibk.ac.at (S.D.)

[3] Atomic and Molecular Collisions Laboratory, Department of Physics, CEFITEC, Universidade NOVA de Lisboa 2829–516 Caparica (Portugal); j.ameixa@campus.fct.unl.pt (J.A).

* Correspondence: Milan.Oncak@uibk.ac.at (M.O); Stephan.Denifl@uibk.ac.at (S.D.).

Table S1 – First ten excitation energies (in eV) of TPZ⁻ calculated at the TD-BMK/aug-cc-pVDZ level in the structure of TPZ optimized at the B3LYP/aug-cc-pVDZ level.

state	E
D_1	1.65
D_2	1.85
D_3	1.95
D_4	2.20
D_5	2.38
D_6	2.58
D_7	2.60
D_8	2.77
D_9	3.06
D_{10}	3.30

Cartesian coordinates (in Ångstrom) and electronic energies calculated at the B3LYP/aug-cc-pVDZ level including the zero-point energy (in Hartree)

```
TPZ
E = -639.643497
C 0.000000 0.784630 0.000000
C -0.452602 2.121181 0.000000
C -1.812753 2.366265 0.000000
C -2.750676 1.303716 0.000000
C -2.320365 -0.009430 0.000000
C -0.936551 -0.275279 0.000000
H 0.291179 2.913778 0.000000
H -2.170264 3.396105 0.000000
H -3.817620 1.523413 0.000000
H -3.008149 -0.850968 0.000000
N -0.463970 -1.598256 0.000000
N 0.831622 -1.854386 0.000000
O -1.269183 -2.555943 0.000000
C 1.698392 -0.825527 0.000000
N 1.346838 0.491241 0.000000
N 3.019435 -1.078579 0.000000
O 2.273073 1.406685 0.000000
H 3.632577 -0.272797 0.000000
H 3.351008 -2.028942 0.000000

O2H
E = -150.922319
O 0.055533 -0.609717 -0.000000
O 0.055533 0.718435 0.000000
H -0.888529 -0.869742 -0.000000

[TPZ-O2H]-
E = -488.725671
C 1.322105 -1.508078 0.002549
C 0.171657 -0.649948 0.001312
C 0.418337 0.759644 -0.000403
C 1.726932 1.278530 -0.002869
C 2.822474 0.425262 -0.003160
C 2.598315 -0.977910 -0.000039
N -0.653793 1.640103 0.003276
N -1.851388 1.195193 0.004261
C -2.103094 -0.226555 -0.000430
N -1.071579 -1.141225 0.002921
N -3.369466 -0.548142 -0.006039
H 1.153022 -2.586302 0.004844
H 3.457607 -1.653948 0.000278
H 3.839340 0.820026 -0.005242
H 1.844617 2.364395 -0.004141
H -3.411359 -1.571342 -0.008434

OH
E = -75.740615
O 0.000000 0.000000 0.108808
H 0.000000 0.000000 -0.870466

N2
E = -109.537259 in
N 0.000000 0.000000 0.552204
N 0.000000 0.000000 -0.552204

[TPZ-HN2O]-
E = -454.436034
N -0.838808 1.807746 -0.000330
C 0.243903 0.960510 0.000235
C -0.193391 -0.403163 -0.000221
N -1.572491 -0.354555 0.000442
C -1.891596 0.972665 -0.000410
C 0.698824 -1.483455 -0.000645
C 2.061245 -1.184298 -0.000155
```

C 2.518485 0.159893 0.000280
C 1.624642 1.232157 0.000287
O -2.406355 -1.386409 0.000497
H 1.980456 2.264564 0.000411
H 3.593430 0.355235 0.000469
H 2.792313 -1.996049 -0.000092
H 0.324943 -2.507215 -0.000798
H -2.933883 1.276537 -0.000967

H2O
E = -76.423418
O -0.000000 -0.000000 0.117802
H -0.000000 0.764237 -0.471206
H -0.000000 -0.764237 -0.471206

N2H
E = -110.039041
N -0.062946 -0.518002 0.000000
N -0.062946 0.661313 0.000000
H 0.881245 -1.003181 -0.000000

[TPZ-H2N2O]-
E = -453.770646
C 0.189705 0.945484 0.000029
C -0.212001 -0.409061 0.000073
C 0.686919 -1.471537 0.000005
C 2.055429 -1.153679 -0.000038
C 2.478559 0.188339 0.000002
C 1.553707 1.247862 0.000041
H 1.885486 2.287880 0.000059
H 3.548729 0.409134 -0.000053
H 2.798481 -1.954036 -0.000102
H 0.334584 -2.503862 0.000006
N -1.609573 -0.348984 -0.000052
O -2.368704 -1.372818 0.000018
C -2.058486 1.088805 0.000018
N -0.930518 1.795578 -0.000068

N3H
E = -164.792644
N 0.295308 1.210424 0.000000
N 0.000000 0.111767 -0.000000
N -0.134151 -1.126358 0.000000
H -1.128096 -1.370839 -0.000000

[TPZ-N3H2O]-
E = -399.138811
C -0.563620 -1.923936 0.000000
C -0.000000 -0.612865 0.000000
C -0.887191 0.554215 0.000000
O -2.143317 0.475049 0.000000
N -0.979068 -3.017390 0.000000
C -0.177537 1.824250 0.000000
C 1.201529 1.916874 0.000000
C 2.022955 0.763652 0.000000
C 1.404856 -0.482886 0.000000
H 2.011329 -1.391631 0.000000
H 3.110250 0.847674 0.000000
H 1.670653 2.906261 0.000000
H -0.798174 2.723211 0.000000

N3
E = -164.155197
N 0.000000 0.000000 -1.184609
N 0.000000 0.000000 -0.000033
N 0.000000 0.000000 1.184642

[TPZ-H2N2O2]-
E = -378.584617
N 1.693066 1.161998 0.000205
C 0.372051 0.721758 -0.000082
C 0.371870 -0.721740 0.000043
N 1.692686 -1.162155 -0.000192
C 2.334621 -0.000173 0.000008
C -0.838655 -1.431800 0.000104
C -2.036965 -0.706624 0.000052
C -2.036799 0.706827 -0.000040
C -0.838479 1.431885 -0.000102
H -0.842180 2.524310 -0.000090
H -2.990480 1.241015 -0.000033
H -2.990654 -1.240777 0.000042
H -0.842819 -2.524243 0.000082

N2O
E = -184.683090
N 1.205931 -0.000069 0.000005
N 0.072642 0.000135 0.000000
O -1.118752 -0.000058 -0.000005

[TPZ-N3H2O2C]
E = -285.644505
C 0.894659 -0.687093 -0.000410
C -0.227128 -1.475042 -0.000001
C -1.470984 -0.688104 0.000036
C -1.471064 0.688661 0.000131
C -0.226319 1.474980 0.000219
C 0.894795 0.686706 -0.000858
H -0.246159 -2.568052 0.000750
H -2.428719 -1.218303 -0.000092
H -2.428698 1.218864 0.000057
H -0.244812 2.568056 0.000483
N 2.140662 -0.000174 0.000587

HOCN
E = -168.644528
C -0.179777 -0.001983 0.000000
O 1.118659 -0.111184 -0.000000
N -1.343462 0.019339 -0.000000
H 1.533622 0.765996 -0.000000

OCN
E = -168.019808
C 0.000000 0.040326 0.000000
N 0.006402 1.271612 0.000000
O -0.005602 -1.142905 -0.000000

N2O-
E = -184.690146

N 1.183422 0.163704 -0.000000
N -0.000000 0.349562 0.000000
O -1.035494 -0.449108 0.000000

[TPZ-N2O]
E = -454.957715
C 0.269893 0.923861 -0.000109
C -0.160790 -0.416904 0.000017
C 1.634561 1.240745 0.000013
C 2.529553 0.172750 0.000076
C 2.089336 -1.171668 0.000006
C 0.733550 -1.488478 0.000003
N -1.571035 -0.415443 0.000043
C -1.969071 0.861646 0.000190
O -2.311962 -1.469754 -0.000009
N -0.874429 1.698353 -0.000501
H -0.920966 2.704876 0.001900
H 0.363382 -2.511622 -0.000046
H 2.828983 -1.971924 -0.000031
H 3.599823 0.379771 0.000146
H 1.984270 2.272640 0.000008
H -3.003743 1.172206 0.000122

CN2-
E = -147.597167
N 1.240410 -0.005156 0.000000
C -0.000000 0.000769 -0.000000
N -1.240410 0.004497 -0.000000

[TPZ-CN2]
E = -492.075296
C -0.000000 0.329183 0.000000
C 1.321202 0.803345 0.000000
C 2.381164 -0.091047 0.000000
C 2.118438 -1.472346 0.000000
C 0.814852 -1.949515 0.000000
C -0.278490 -1.062689 0.000000
H 0.598520 -3.016864 0.000000
H 2.946255 -2.182292 0.000000
H 3.405752 0.277410 0.000000
H 1.479557 1.878766 0.000000
N -1.063987 1.315672 0.000000
O -0.762944 2.526015 0.000000
O -1.511733 -1.586216 0.000000
N -2.271148 0.871805 0.000000
H -2.883271 1.693174 0.000000
H -2.146443 -0.822505 0.000000

CN-
E = -92.868847
N 0.000000 0.000000 0.546164
C -0.000000 0.000000 -0.637191

[TPZ-CN]
E = -546.753275
C -1.959609 -0.064566 0.000110
C -0.658935 0.483145 -0.000018
C 0.449110 -0.411706 -0.000060
C 0.236317 -1.794620 -0.000102
C -1.054424 -2.312860 -0.000048
C -2.152395 -1.438356 0.000070
N -0.526134 1.868574 -0.000063
O -1.525888 2.657256 -0.000040
N 1.842830 0.023522 0.000058
O 2.732058 -0.854962 0.000032
N 2.072492 1.284390 0.000056
H -2.795631 0.630554 0.000203
H -3.167474 -1.835977 0.000148
H -1.204713 -3.391451 -0.000143
H 1.108490 -2.442524 -0.000072
H 3.093936 1.379917 0.000059
H 0.431340 2.239507 -0.000193

[TPZ-CN3]
E = -437.334246
C 2.070440 -1.009521 -0.000000
C 0.756828 -1.460142 0.000049
C -0.310743 -0.540240 0.000095
C -0.051057 0.857648 0.000146
C 1.280909 1.290612 -0.000053
C 2.329256 0.371505 -0.000102
N -1.616351 -1.017433 0.000034
H -1.766915 -2.027218 0.000175
O -1.044081 1.770339 0.000103
H -1.895757 1.270533 -0.000055
H 0.536740 -2.529464 0.000031
H 2.891499 -1.725136 -0.000006
H 3.357956 0.731673 -0.000239
H 1.468222 2.363778 -0.000157
O -2.672306 -0.273002 -0.000202

O-
E = -75.138730 in
O 0.000000 0.000000 0.000000

[TPZ-O]
E = -564.475108
C 1.482135 -1.699783 0.004563
C 0.321069 -0.881257 0.001902
C 0.515418 0.523667 -0.002216
C 1.795271 1.110227 -0.004975
C 2.902265 0.282791 -0.003653
C 2.738098 -1.125545 0.001482
N -0.642328 1.332882 0.001275
N -1.844991 0.801443 0.000527
C -1.917561 -0.568160 -0.002864
N -0.918979 -1.436227 0.004404
N -3.195387 -1.037314 -0.034832
H 1.344281 -2.779899 0.007917
H 3.621173 -1.765527 0.002724
H 3.903150 0.712852 -0.006290
H 1.878340 2.194001 -0.007954
H -3.345664 -2.026049 0.088836
H -3.958622 -0.391777 0.093360
O -0.531379 2.572409 0.007044

NH2-
E = -55.899592

N -0.000000 0.000000 0.144578
H -0.000000 0.804290 -0.506022
H -0.000000 -0.804290 -0.506022

[TPZ-NH2]
E = -583.700141
C -1.520461 -1.656491 0.000008
C -0.315228 -0.886353 0.000032
C -0.435511 0.534371 0.000044
C -1.665985 1.172658 0.000031
C -2.826443 0.387308 0.000014
C -2.749197 -1.022402 0.000002
N 0.787607 1.297346 0.000021
N 1.946449 0.722370 0.000056
C 2.000745 -0.692738 0.000324
N 0.868665 -1.508097 0.000013
O 0.690930 2.536084 -0.000030
H -1.424341 -2.741154 -0.000007
H -3.666044 -1.611481 -0.000018
H -3.800586 0.875732 0.000004
H -1.704734 2.259289 0.000032
O 3.115212 -1.208816 -0.000391

[TPZ-N2O2]
E = -379.788418
C -0.951728 -1.430435 -0.000091
C 0.266977 -0.733023 -0.000062
C 0.264815 0.687599 0.000070
C -0.917078 1.437636 0.000150
C -2.114965 0.723946 0.000030
C -2.132770 -0.689246 -0.000079
N 1.600136 1.040349 -0.000068
C 2.319018 -0.123640 -0.000002
N 1.573461 -1.209601 0.000180
H -0.962231 -2.520442 -0.000059
H -3.094269 -1.207082 -0.000104
H -3.060016 1.270367 -0.000005
H -0.902304 2.528017 0.000183
H 1.992515 1.983557 -0.000912
H 3.405527 -0.106674 0.000013

O2
E = -150.345987
O 0.000000 0.000000 0.604274
O 0.000000 0.000000 -0.604274

[TPZ-H2O]-
E = -563.276150
C -2.196522 0.377898 -0.000000
C -0.770067 0.556638 -0.000000
C -0.000000 -0.648651 -0.000000
C -0.577638 -1.925507 -0.000000
C -1.960485 -2.053784 -0.000000
C -2.760855 -0.881209 -0.000000
N 1.413750 -0.494076 0.000000
N 1.976878 0.668233 0.000000
C 1.144651 1.822777 0.000000
N -0.231415 1.776979 0.000000
O 2.132049 -1.536216 0.000000
N 1.793679 2.953101 0.000000
H -2.810719 1.278647 -0.000000
H -3.849587 -0.977690 -0.000000
H -2.424237 -3.040390 -0.000000
H 0.083403 -2.789457 -0.000000

Cartesian coordinates (in Ångstrom) and electronic energies calculated at the CASSCF(3,5)/6-31g* (in Hartree)

```
D1MIN
E(D1) = -635.87443121
C 0.0432996284 0.7708945120 -0.0112642630
C -0.4404683431 2.1110242147 0.0769624468
C -1.7678026241 2.3477412342 0.0686559725
C -2.7259441316 1.2679610416 -0.0170044520
C -2.2914602152 -0.0106422713 -0.0822322643
C -0.9078907927 -0.2980300386 -0.1026083564
H 0.2847132357 2.8978977106 0.1364073080
H -2.1276372562 3.3612470254 0.1304272143
H -3.7787326270 1.4889809637 -0.0214224492
H -2.9734637365 -0.8368590027 -0.1180106083
N -0.4413714154 -1.5915686862 0.0169892811
N 0.7898043551 -1.8632334664 -0.0110146520
O -1.2615450132 -2.5097510770 0.1981391118
C 1.6846204509 -0.8290055861 -0.3584583472
N 1.3411544094 0.4804700730 -0.0697173139
N 3.0347954515 -1.1908026045 -0.1878985254
O 2.2674144075 1.3777084642 0.0210456838
H 3.5639300167 -0.3410351652 -0.1538085474
H 3.1485751993 -1.6760903413 0.6848127606

D0/D1 CI
E(D0) = -635.874406907115
E(D1) = -635.874381755510
C  0.7463223462 -0.0167301058 0.3176613762
C  1.7586239704 -0.0966861130 1.3221906947
C  1.4172890501 -0.0458699418 2.6247160644
C  0.0358944732 0.0767563596 3.0391314441
C  -0.9407937182 0.1338680827 2.1073810672
C  -0.6216367149 0.1126774206 0.7298971346
H  2.7759025302 -0.1835630548 0.9961455269
H  2.1850162747 -0.1010471888 3.3784243994
H  -0.2041905079 0.1143845018 4.0870770298
H  -1.9760031800 0.1947817146 2.3791042829
N  -1.6017294673 -0.0309499206 -0.2313327746
N  -1.3316862568 -0.0436422579 -1.4625888021
O  -2.7792305979 -0.1947186604 0.1332341120
C  -0.0143588184 0.2928102845 -1.8488621461
N  1.0276927431 0.0050774956 -0.9820795941
N  0.2197531020 0.0873044270 -3.2222393834
O  2.2276055787 -0.1180709023 -1.4457740583
H  1.2123848565 0.0366655215 -3.3463317712
H  -0.1850838723 -0.7871250288 -3.5079354574
```
